# Serum Biochemistry of Lumpy Skin Disease Virus-Infected Cattle

**DOI:** 10.1155/2016/6257984

**Published:** 2016-05-12

**Authors:** Murat Şevik, Oğuzhan Avci, Müge Doğan, Ömer Barış İnce

**Affiliations:** ^1^Department of Molecular Microbiology, Veterinary Control Institute, Meram, 42080 Konya, Turkey; ^2^Department of Virology, Faculty of Veterinary Medicine, Selçuk University, 42000 Konya, Turkey; ^3^Agriculture and Rural Development Support Institution, 03000 Afyonkarahisar, Turkey

## Abstract

Lumpy skin disease is an economically important poxvirus disease of cattle. Vaccination is the main method of control but sporadic outbreaks have been reported in Turkey. This study was carried out to determine the changes in serum biochemical values of cattle naturally infected with lumpy skin disease virus (LSDV). For this study, blood samples in EDTA, serum samples, and nodular skin lesions were obtained from clinically infected animals (*n* = 15) whereas blood samples in EDTA and serum samples were collected from healthy animals (*n* = 15). A quantitative real-time PCR method was used to detect* Capripoxvirus* (CaPV) DNA in clinical samples. A real-time PCR high-resolution melt assay was performed to genotype CaPVs. Serum cardiac, hepatic, and renal damage markers and lipid metabolism products were measured by autoanalyzer. LSDV nucleic acid was detected in all samples which were obtained from clinically infected cattle. The results of serum biochemical analysis showed that aspartate aminotransferase, alkaline phosphatase, total protein, and creatinine concentrations were markedly increased in serum from infected animals. However, there were no significant differences in the other biochemical parameters evaluated. The results of the current study suggest that liver and kidney failures occur during LSDV infection. These findings may help in developing effective treatment strategies in LSDV infection.

## 1. Introduction

Lumpy skin disease (LSD) is a viral disease of cattle caused by lumpy skin disease virus (LSDV). The causative agent is a member of the* Capripoxvirus* genus in the Poxviridae family [1]. LSDV has double-stranded DNA genome, which encodes 30 homologues of poxviral proteins known to be structural or nonstructural, and it is antigenically and genetically closely related to sheeppox virus (SPPV) and goatpox virus (GTPV) with nucleotide sequence identities of 96% between species [2, 3].

Clinical disease is seen in cattle and wildlife animals such as the Arabian oryx and water buffalo, but LSDV does not naturally infect sheep and goats [4–6]. The disease is characterized by fever, nodules (2 to 5 cm in diameter) on the skin and mucous membranes, lesions in the respiratory and gastrointestinal tracts, and enlarged superficial lymph nodes. It has an important economic impact on the cattle industry due to loss in milk production and condition, infertility, abortion, damaged hides, and sometimes death because of secondary bacterial infections [7–10]. Mortality rates in naive population of cattle may reach 5% whereas morbidity rates vary from 3% to 85% [11–13]. It is thought that LSDV is transmitted among cattle by biting insects (such as mosquitoes, flies, and ticks) [14, 15].

LSD has been reported in most countries in Africa, the Middle East including Egypt, Lebanon, Jordan, Israel, Iran, Iraq, and Turkey, and Central Asia (Azerbaijan). The disease was also reported in Cyprus, Greece, and the Russian Federation [16–21]. There is a potential risk that LSDV could spread from Middle East, further into Europe because of global climatic changes, vectors, and trade movement in animals and animal products.

Clinical lesions can be confused with bovine herpes virus 2 (Allerton) infections, insect bites, dermatophilosis, and bovine besnoitiosis [8]. Therefore, laboratory confirmation is needed. Laboratory diagnosis of LSD can be performed by using serological and molecular techniques and by virus isolation in cell cultures [22].

Pathogenic mechanism of viral disease involves implantation of virus at the portal of entry, replication at that site, spread to target organs, and spread to sites of shedding of virus into the environment. Viral disease occurs if the virus replicates in essential cells sufficiently and destroys them directly or damages organ function indirectly as a result of the host immune response to the presence of virus proteins [23]. Significant changes can be observed in serum biochemical values when cellular/organ damage occurs. There have been few studies conducted on pathogenesis of LSD in cattle [24–27]. Additionally, there is limited information in the literature about the serum biochemical findings of cattle naturally infected with LSDV. Serum biochemical parameters can be a useful tool for assessing animal health and help better understanding the pathogenesis of the disease. The purpose of the study therefore was to investigate the changes in serum biochemical values of cattle infected with LSDV.

## 2. Material and Methods

### 2.1. Samples

In the first stage, a list of villages in Konya Province where LSD was previously reported was obtained from the district livestock office and then a recently infected farm was determined. The sampled farm was a large sized farm (*n* > 50 cattle) which voluntarily joined to this study. The farm owner reported that there was a marked reduction in milk production (40% less milk) and that cattle had fever (above 40°C) for more than two days. Skin lesions and lesions in the mucous membranes of the mouth, lacrimation, nasal discharge, anorexia, unwillingness, emaciation, and enlarged lymph nodes were observed in infected animals. Skin lesions (2 to 6 cm in diameter) occurred mostly in the area of the neck and back. Mammary gland and teats lesions were also observed in some of the cows. Nodular skin lesions, swabs, and blood in EDTA were taken from clinically ill cattle (*n* = 15). Blood samples (5 mL) were obtained from the jugular vein of each animal by atraumatic jugular venipuncture using vacutainer tubes. Sampled cattle were Brown Swiss and aged between 1 and 3 years (median age 1.9 years). Whole blood samples with and without anticoagulant were also taken from healthy cattle (*n* = 15; Brown Swiss and aged between 1 and 3 years; median age 1.8 years) originating from a control farm where LSD was not reported. Control animals had body temperatures between 38.1°C and 38.5°C and no disease was reported on this farm. Cattle were also not recently vaccinated against any disease. Control cattle were free of any external, blood, and internal parasites. Furthermore, there were no different management practices applied in sampled and control farms.

Whole blood samples without anticoagulant were centrifuged at 3000 rpm for 10 min for serum separation and stored at −20°C until testing. Buffy coats were extracted from EDTA blood by using PBS with 2% v/v foetal bovine serum (FBS) and stored at −20°C until PCR analysis.

### 2.2. DNA Extraction from Clinical Samples

DNA was extracted from buffy coat cells, swabs, and sheep and goat pox vaccine using a MagNA Pure LC 2.0 system (Roche Applied Science, Indianapolis, IN, USA) with the Magna Pure LC total nucleic acid isolation kit (Roche Applied Science, Indianapolis, IN, USA). For skin lesions, samples were processed as 10% (w/v) homogenates in viral transport medium, prepared using a tissue Ruptor (Qiagen, Hilden, Germany). DNA was extracted from the skin homogenates using the QIAamp cador Pathogen Mini Kit (Qiagen, Hilden, Germany) according to the manufacturer's instructions.

### 2.3. Quantitative Real-Time PCR

A quantitative real-time PCR was performed using a minor grove binding TaqMan probe which amplifies a 89 bp fragment of the CaPV open reading frame (ORF) 074 [25]. The assay was carried out in a 25 *μ*L reaction mixture containing 5 *μ*L of the extracted DNA using the LightCycler 480 Instrument II real-time PCR machine (Roche Applied Science, Indianapolis, IN, USA). The amplification conditions used were 50°C for 2 min, 95°C for 10 min, 45 cycles of 95°C for 15 s, and 60°C for 1 min. Lyophilized freeze-dried live sheep and goat pox vaccine obtained from the Pendik Veterinary Control Institute, Istanbul, Turkey, was used as the positive control.

### 2.4. Genotyping of CaPVs

The real-time PCR high-resolution melt (HRM) assay targeting the RPO30 gene of CaPVs was used as previously described [28] for genotyping of field samples which were found positive by real-time PCR assay. GTPV Turkey/2015 Nigde, SPPV Turkey/2015-Akşehir, and LSDV Turkey/2014-01 strains obtained from the Konya Veterinary Control Institute (Konya, Turkey) were used as positive controls.

### 2.5. Analysis of Serum Biochemical Parameters

Biochemical analyses were performed using an autoanalyzer (ILab-300 Biomerieux Diagnostic, Milan, Italy). Creatine kinase-myocardial band (CK-MB), alanine aminotransferase (ALT), aspartate aminotransferase (AST), alkaline phosphatase (ALP), blood urea nitrogen (BUN), gamma glutamyltransferase (GGTS), total protein (TP), albumin, creatinine, and cholesterol levels were analyzed.

### 2.6. Statistical Analysis

The results were expressed as mean ± standard deviations. Differences between the LSDV-infected and healthy groups were calculated by using the two-sample *t*-test. *P* < 0.05 was considered to be statistically significant. All statistical analysis was performed with SPSS 19.0 (SPSS Inc., Chicago, IL, USA).

## 3. Results

### 3.1. Detection of Viral DNA by Real-Time PCR

Viral DNA was detected in all suspected animals (*n* = 15) and was negative in all control animals (*n* = 15). The cycle threshold (Ct) values of positive skin nodules and vesicle swabs were in the range of 16 to 26, and EDTA whole blood samples were in the range of 25 to 36.

### 3.2. Real-Time PCR-HRM Assay for Genotyping CaPVs

Real-time PCR-HRM assay confirmed that all of the positive samples were LSDV.

### 3.3. Serum Biochemical Values of LSDV-Infected and Uninfected Cattle

Serum biochemical values obtained from cattle naturally infected with LSDV and healthy cattle are given in Table 1. Whereas CK-MB, ALT, BUN, GGTS, albumin, and cholesterol levels were not significantly altered in infected cattle, the creatinine and total protein concentrations as well as the serum AST and ALP activities were markedly increased (*P* < 0.05). The ALT, blood urea nitrogen, and albumin levels were increased slightly but not significantly compared to the control values.

## 4. Discussion

Lumpy skin disease is an important transboundary disease of cattle and has recently spread out of Africa into Europe [20, 29, 30]. The disease causes considerable devastating economic losses mainly due to permanent hide damage, milk production, abortion and infertility, and emaciation and disruption in the trade of cattle and their products [22, 29].

Serum biochemical references of cattle naturally infected with LSDV are scanty. The measurement and evaluation of the biochemical profile may be helpful in elucidating the pathogenesis and prognosis of the disease. Significant changes occur in the blood biochemical parameters of the animals exposed to viral diseases [31–33]. It has been reported that elevated serum cardiac troponin I is associated with myocardial injury during foot-and-mouth disease virus infection [34]. Krey et al. announced that low-density lipoprotein is a natural receptor for bovine viral diarrhoea virus [35]. Pani et al. reported that abnormal accumulation of cholesterol esters could be used as an indicator of susceptibility to prion infection [36]. Additionally, Nikolay et al. reported that changes in the level of serum ALT, AST, and total protein could be used as prognostic markers of the course of the bovine leukaemia virus infection [32]. Biochemical indicators can be helpful in understanding the course of the disease. Therefore, this study was designed to investigate changes that may occur in serum enzyme activities in cattle infected with LSDV.

LSDV-infected cattle in the current study had significantly higher AST concentrations and a slightly insignificant increase in ALT was determined in infected cattle. An insignificant increase in the rate of ALT can be explained by the low ALT activity in cattle liver cells [37]. It was reported that the increased AST concentration in the serum is a sensitive indicator of hepatocyte damage, even if the damage is of a subclinical nature, and in monitoring its progress [38–40].

AST besides being present in the hepatocytes is also present in skeletal muscle and muscular cardiac cells. This increase can be related to the cardiac injury produced by the presence of the virus in the heart [41]. A slightly insignificant increase in CK-MB was determined in infected cattle. CK-MB is another cardiac biomarker that has been reported to increase with myocardial damage [42, 43]. However, it was reported that CK-MB is not suitable for diagnosing myocardial injury because significant amount of the CK-MB isoenzyme is found in skeletal muscle. Therefore, skeletal muscle injury may contribute to an increase in the absolute activity of CK-MB fraction in blood [44, 45]. Measuring cardiac troponin I would have helped in the diagnosis of myocardial injury. Unfortunately, it was not done in this study. LSD lesions can occur in the muscle fascia and in the muscle itself [8]. Increased AST concentrations in infected cattle can also be related to muscular injuries [46].

ALP is an enzyme in the cells lining the biliary ducts of the liver, and its concentration increases with biliary disease, intrahepatic cholestasis, and infiltrative diseases of the liver [47]. We determined a significant increase in the level of ALP concentrations in infected cattle, which seems contrary to previous study that suggests that ALP concentrations are not changed in LSDV-infected cattle [48]. The differences between our and previous result may be related to the stage of the infection and the age of animals. It has been reported that both ALP and GGT levels are elevated in cholestasis [49]. In this study, no significant GGT differences were observed between infected and control groups. Therefore, the increase in the level of AST, ALT, and ALP in LSDV-infected cattle can be related to the hepatic injury produced by the presence of the virus in the liver. This finding is in agreement with a previous study that reported that pox lesions can be seen in the liver [25].

It has been reported that an increase in creatinine concentration reflects a reduction in the glomerular filtration rate [50, 51]. Serum concentration of creatinine was significantly higher in the LSDV-infected group than in the control group. On the contrary, Abutarbush [48] found low creatinine concentrations in cattle naturally infected with LSDV. Possible explanations for this discrepancy may be the time of sampling, techniques used for serum biochemical analysis, and individual differences. It has been reported that absolute muscle mass and level of physical activity may influence the rate of creatinine production and thus the serum concentration [52]. Additionally, effects of age, breed, gender, diet, heat stress, lactation, and pregnancy period on serum creatinine levels have been documented [53–56]. Cattle used in this study were Brown Swiss and aged between 1 and 3 years whereas cattle used in Abutarbush's study were Holstein-Friesian and the age of the animal ranged from 5 months to 10 years. Furthermore, differences in creatinine concentration between these two studies can be explained by differences in muscle mass and diet of the cattle and physical activity.

The results of the present study revealed that total serum protein concentrations in cattle positive for LSD were increased and significantly higher than those of healthy cattle. Furthermore, a slightly insignificant increase in albumin was determined in infected cattle. This is expected because it indicates an activation of immune response following infection. It has been reported that changes in total protein and albumin concentrations are connected with humoral immune response to infectious pathogens [57, 58].

In the present study, BUN concentrations were enhanced in LSDV-infected cattle. Also, albumin concentrations rose slightly along with BUN in infected cattle (Table 1). This can be explained by the dehydration of cattle [59]. Fever, anorexia, and lethargy are commonly observed clinical signs in LSDV-infected cattle. Therefore, dehydration can develop after the onset of the clinical signs produced by LSD.

## 5. Conclusions

Not enough published data are available for the serum biochemistry of LSDV-infected cattle. As a conclusion, blood biochemistry analyses suggest that LSD causes liver and renal damage in cattle. This study provided information to a better understanding of the pathogenesis of the disease, and findings of the study may give further insight to improve treatment strategies in LSDV infection.

## Figures and Tables

**Table 1:** Mean ± SD of the biochemical profiles in LSDV infected cattle and in healthy cattle.

Parameters	LSDV infected group	Control group	*P* values
CK-MB (UI)	254.9 ± 52.44	204.5 ± 64.11	*P* = 0.07
ALT (U/L)	28.6 ± 17.38	26.6 ± 1.71	*P* = 0.721
AST (U/L)	89.5 ± 32.5	67.4 ± 4.6	*P* = 0.047
ALP (U/L)	85.2 ± 82.12	32.8 ± 3.66	*P* = 0.047
BUN (mg/dL)	16.1 ± 9.16	10.2 ± 1.8	*P* = 0.060
GGT (U/L)	21.3 ± 8.54	24.5 ± 8.85	*P* = 0.421
TP (g/dL)	7.14 ± 1.13	6.22 ± 0.48	*P* = 0.028
Albumin (g/dL)	3.68 ± 0.87	3.24 ± 0.26	*P* = 0.144
Creatinine (mg/dL)	0.9 ± 0.26	0.62 ± 0.11	*P* = 0.007
Cholesterol (mg/dL)	127.8 ± 53.35	129.2 ± 31.7	*P* = 0.945
